# Reply to Veitia

**DOI:** 10.1038/s41431-020-00802-9

**Published:** 2021-01-18

**Authors:** Marcus Danielsson, Jonatan Halvardson, Jonas Mattisson, Lars A. Forsberg

**Affiliations:** 1 Department of Immunology, Genetics and Pathology, Science for Life Laboratory, Uppsala University, Uppsala, Sweden; 2 The Beijer Laboratory, Uppsala University, Uppsala, Sweden

**Keywords:** Genetics, Biomarkers

## To the Editor:

Establishment of common methods is an advantage for comparability of studies. We have read the insightful Correspondence in EJHG in which Veitia addresses the performance of two recently published formulas for transforming intensity signals from SNP-array data of mosaic loss of chromosome Y (LOY) into the percentage of cells with LOY in a sample. Acquired loss of chromosome Y is the most common somatic mutation in human blood cells [1–8] and affected men have an increased risk for all-cause mortality [1, 5]. Furthermore, LOY in blood has been found to be associated with risk for various human disease such as hematological and non-hematological cancers, autoimmune conditions, Alzheimer’s disease, cardiovascular diseases, schizophrenia, diabetes, and age-related macular degeneration [1–3, 5, 8] (and references therein). In addition to age, replicated risk factors associated with LOY in blood include smoking [4, 5, 9] and genetic susceptibility [4, 6].

LOY mosaicism has been quantified using technologies such as karyotyping, qPCR, SNP-arrays, droplet digital PCR (ddPCR), and next generation sequencing. A commonly used method to estimate the level of LOY is calculation of the mLRRY [1] from data generated by SNP arrays as the median Log R Ratio of probes located in the male specific part of chromosome Y. Since the mLRRY is non-linear and on an inversed scale, different formulas has been suggested recently for the transformation of individual mLRRY values into the proportion of cells with LOY in a sample, to increase the comparability of readouts from SNP-arrays with other methods. Thus, we recently published in *EJHG* [8] the empirically derived formula LOY(%) = 100(1 − 2^(2*mLRRY)^) describing the relationship between mLRRY from Illumina arrays and LOY(%) in blood samples. This formula (referred to as Danielsson’s) was established by comparing LOY-estimations in matched DNA samples that were analyzed using SNP arrays, whole-genome sequencing and ddPCR. A strength of this formula is that it returns the LOY(%) in the range of zero to 100% of affected cells with a considerable concordance with LOY-readouts using independent methods (for example see Fig. 3 in [8]). We also compared the performance of Danielsson’s formula with a previously suggested formula published by Veitia and colleges [10]. Our results showed that Veitia’s formula generated unrealistic estimates in samples with high levels of LOY (see Fig. 4 in [8]).

In the correspondence, the accuracy and usefulness of these two formulas are discussed. As recognized also by Veitia, the two formulas generate similar estimates of LOY(%) in samples with low-level mosaicism. Nevertheless, a comparative analysis indicated that Veitia’s formula would perform marginally better in such samples. It should be noted, however, that this analysis was performed with an incomplete dataset that was extracted from Fig. 2a and b in [8] using the WebPlotDigitizer tool. Thus, even though the full dataset was available in Supplementary Table 3 in [8], the analyzed data omitted one data point in Fig. 2a and included only 71 of the 121 data points in Fig. 2b. It is unclear if and how this limitation in Veitia’s analysis biased the results and conclusions regarding the relative accuracy of the two formulas. Yet, it is suggested in the Correspondence that Veitia’s formula should be preferred for analysis of samples with low-level LOY mosaicism and that Danielsson’s should be used for samples with higher level of mosaicism.

This discussion is appreciated and we agree that it is important to establish guidelines within this field. We however disagree with the suggestion to implement different formulas for samples with low and high levels of LOY for several reasons. It should first be emphasized again that the two formulas generate highly comparable estimates of LOY(%) in samples with low-level mosaicism. Regardless of the statistically significant result in Veitia’s comparative analysis of the two formulas, it should be noted that the biological relevance of this result is rather insignificant. Importantly, as illustrated in Fig. 3 in [8] comparing results from three independent technologies, it is clear that Danielsson’s formula accurately transform mLRRY values to LOY(%) over the entire spectra of mosaicism, from zero to 100% of cells affected with LOY. Linear regression analyses displayed *R*^2^ values of 0.965 and 0.959 for comparisons between SNP-array/WGS and SNP-array/ddPCR, respectively. These results show that Danielsson’s formula is sufficient for transformation of mLRRY into LOY(%) also in samples with low level of LOY mosaicism. In contrast, as mentioned above, Veitia’s formula yields unrealistic estimates of LOY(%) in samples with higher levels of LOY mosaicism, even beyond the theoretical maximum of 100%. In this context, Veitia reasons that samples with high levels of LOY mosaicism “concerns a few data points” that would represent an “exception rather than the rule”. However, recent large population studies of normally aging men suggest the opposite; a considerable proportion of aging men are indeed affected with LOY in at least 50% of peripheral blood cells (for example see Fig. 1a in [4]).

In conclusion, we argue that the suggestion to use different formulas for samples with low and high levels of LOY mosaicism adds unnecessary complexity without providing substantial additional value. Furthermore, the comparability of studies in the field would benefit from adopting the same formula for estimating LOY(%) from SNP-array data. As a final point, it is important to consider that Danielsson’s formula was optimized for mLRRY data generated from Illumina’s SNP-arrays. Further studies are needed to evaluate similar formulas for LOY(%) transformations from intensity data generated by DNA-arrays from other manufacturers.
